# Smart-parking management algorithms in smart city

**DOI:** 10.1038/s41598-022-10076-4

**Published:** 2022-04-20

**Authors:** Mahdi Jemmali, Loai Kayed B. Melhim, Mafawez T. Alharbi, Abdullah Bajahzar, Mohamed Nazih Omri

**Affiliations:** 1 Department of Health Information Management and Technology, College of Applied Medical Sciences, University of Hafr Al Batin, Hafr Al Batin, 39524 Saudi Arabia; 2 Department of Computer Science and Information, College of Science in Zulfi, Majmaah University, AL-Majmaah, 11952 Saudi Arabia; 3 MARS Laboratory, University of Sousse, Sousse, Tunisia; 4 Department of Computer Science, Higher Institute of Computer Science and Mathematics, Monastir University, 5000 Monastir, Tunisia; 5 Department of Natural and Applied Sciences, Applied College, Qassim University, Buraydah, Saudi Arabia

**Keywords:** Computer science, Information technology

## Abstract

Recently, various advanced technologies have been employed to build smart cities. Smart cities aim at improving the quality of life through the delivery of better services. One of the current services that are essential for any smart city, is the availability of enough parking spaces to ensure smooth and easy traffic flow. This research proposes a new framework for solving the problem of parking lot allocation, which emphasizes the equitable allocation of people based on the overall count of people in each parking space. The allocation process is performed while considering the available parking lots in each parking space. To accomplish the desired goal, this research will develop a set of seven algorithms to reduce the gap in the number of people between parking spaces. Many experiments carried out on 2430 different cases to cover several aspects such as the execution time and the gap calculations, were used to explore the performance of the developed algorithm. Analyzing the obtained results indicates a good performance behavior of the developed algorithms. Also, it shows that the developed algorithms can solve the studied problem in terms of gap and time calculations. The MR algorithm gained excellent performance results compared to one of the best algorithms in the literature. The MR algorithm has a percentage of 96.1 %, an average gap of 0.02, and a good execution time of 0.007 s.

## Introduction

### Context

Smart cities have public spaces such as parks, zoos, playgrounds for entertainment and commercial areas for shopping and business activities^1–5^. To facilitate traffic easy flow to these locations, technologies, such as motion sensors and cameras were used. In addition, traffic personnel is employed to regulate the entry and the exit of vehicles, to and from the available parking lots, in the assigned parking spaces. Despite these efforts and these means put in place, many problems remain inevitable when directing different vehicles to the available parking lots and often cause congestion of visitors at the entrance doors to the desired spaces; depending on the number of people in each vehicle which may vary from one vehicle to another. The first problem in this scenario is the accumulation of vehicles when entering and leaving parking spaces, which causes a blockage in traffic inside these parking spaces. The second problem is that of the long waiting time, by the passengers inside each vehicle, during the search for the available parking lot in the assigned parking space^6,7^.

For the vehicles’ passengers, this waiting time is an important factor that can be decisive, hence its consideration becomes a necessity. Indeed, this factor is a direct cause of the reluctance of new visits by the same visitors in the future, which leads to a decrease in the number of visitors to these places. Consequently, this decrease has a direct and negative impact on the financial returns of many companies. Thus, attempting to manage these parking spaces by conventional methods may generate significant congestion of visitors at the entrance gates to these places because of the different number of people in each vehicle.

### Goals and contributions

In this work, the proposed solution is initiated by dividing each parking space into a set of known and identified parking lots. This division involves optimizing the allocation of vehicles in the available parking spaces, using new techniques (entity rules, for example) based on the overall number of people in each parking space, and not on the fair distribution of vehicles between parking spaces. The given solution can be adopted by different types of parking spaces such as airports, shopping centers, public parks, entertainment venues, and many other areas that require optimal management of the available parking spaces.

### Paper structure

The remainder of this paper is organized as follows. The coming section starts by presenting an overview of existing works on parking management problems for smart cities, in this context the research gaps will be discussed in “Research gaps” section. “Problem formulation” section deals with the formulation of the presented problem. Then, “The developed algorithms” section, introduces the proposed approach as heuristics. Experimental setup is explained in “Experimental results and discussion” section, which includes the used environment, materials and the required data set. Moreover, this section introduces the evaluation tools that will be considered as the evaluation metrics of the developed algorithms. Also, this section presents and discusses the experimental results. The last section, which is “Experimental results and discussion” section displays the extracted conclusions and suggests some of the future directions.

## Related work

In several previous studies, many researchers have applied different strategies and techniques to address the problem of assigning vehicles to available parking spaces. For example, the problem of allocating available parking spaces to drivers based on driver preferences was discussed in^8^ where the authors proposed a parking system that allows the user to reserve a parking space closest to the place they will visit. This approach helps ensure more efficient use of parking spaces. The same concept was introduced in^9^ where the authors used the negotiation of software agents to present an approach for reserving available parking spaces in terms of user preferences, taking into account the preferences of parking space owners. In an attempt to handle the problem of parking allocation in urban areas, the authors of^10^ used private parking lots available during the day by sharing indoor parking spaces between inhabitants and other users to meet demands that other parking requests. The proposed framework is suitable for urban parking spaces and can be a good solution to overcome the lack of parking spaces. However, this solution cannot be adapted in our case due to the different types of parking that will be considered in the context of this work. In^11,12^ the authors developed several parking policies through a framework that uses vehicle GPS data to solve the dynamic parking allocation problem by treating a set of 0-1 programming models. Power consumption is critical for hybrid vehicles, which has motivated the authors in^13^ to use the parking allocation problem to solve the charging port distribution problem for hybrid and electric cars in the aim to minimize the cost of the total power of the system. In another work^14^ the authors presented an online parking space reservation feature that gives users the option of reserving their parking space in advance. This approach ensures a parking space for each vehicle and also helps to get the most out of parking spaces for cars. However, it does not help alleviate the occurrence of traffic jams, which leads to higher energy consumption and longer waiting time to reach the reserved parking space. Managing available parking lots in central commercial areas was discussed by^15^. The authors presented a Genetic Algorithm method with Dynamic Shared Parking to generate the approximately optimal solution, where the drivers in this method rent idle parking lots that are nearby their destinations based on cost, distance and time limitations. This method is limited to certain vehicles type and does not consider the number of passengers in every vehicle. Using the patterns of driver’s arrival and departure time distributions, the authors in^16^ presented Chance-constraint optimization, to address the shared-parking allocation problem. The objective was to increase the extent of parking utilization level and reduce the service failure rate. The authors in^17^ Presented an online resource allocation problem, for urban parking management by proposing a multi-agent system that considers the dynamic geographical positions and the nondeterministic online appearance for drivers and parking spots. The multi-agent system relied on the information that could be obtained from groups of drivers about the available parking spaces to minimize the required searching time to locate a valid parking spots to drivers. This approach is specially dedicated to the problem of static parking allocation and cannot handle the real-time changes in the available parking spaces i.e. the dynamic parking allocation problem. Thus, it will not prevent vehicles overcrowding in parking spaces.

To avoid traffic jams when finding a parking spot and to allow drivers to easily locate available parking spots, the authors of^18^ used driver preferences to come up with a parking system that works with a dynamic parking allocation problem based on an online multi-agent approach. This approach is based on a shortest route guidance module to help the driver reach the reserved parking space. Likewise, the authors in^19^ presented the Reservation-based Smart Parking System (RSPS) that uses cluster based algorithm to handle the dynamic parking allocation problem. The proposed system deploys a set of sensor wireless networks to update the list of available parking spots, which enables the driver to use their personal smart phones to identify and reserve the nearest free pots in the parking spaces. The proposed system is said to simplify the parking process, in addition to alleviating traffic congestion resulted from the searching for parking spot. Another work from^20^ has also been proposed, in which the authors have developed a prediction-based parking allocation framework that combines occupancy prediction and parking allocation to provide users with parking services. Besides, the authors in^21^ discussed online parking assignment for connected vehicles and non-connected vehicles by proposing a multi-agent deep reinforcement learning framework to handle challenges caused by the uncertain availability of parking lots due to reservations performed by non-connected vehicles. Allocating of parking spaces at hospitals was the goal of the cumulative model which was built based on prospect theory to address allocation issues for shared parking space problem presented by^22^ to handle all challenges regarding the lack of parking lots facing patients and hospital visitors with the objective of maximizing the returns of the available parking lots based on the users’ choice under time window constraints.

The authors in^23^ applied agile algorithm to increase the effectiveness of existing cloud-based parking system and employed IOT technology to present a network architecture. This architecture was used to produce a system that assigns the available parking lots based on the vehicles size while ensuring low cost and minimum waiting time. The presented system addresses the reservation of parking lot with load balancing and congestion avoidance based on the dimensions and the available of parking lots for each parking space. Moreover, the proposed system can suggest some solution based on the user references, if the selected parking space was full with the option of forwarding the driver to the new available parking lot. Although the research presented by^23^ addressed the issues of load balancing, minimizing waiting time and congestion avoidance, which make the presented system a near optimal solution for the parking allocation problem ; it did not consider different number of passengers in each vehicle and the overall number of people inside each parking space if it was fully utilized. Moreover, the load balancing here was based on the overall number of vehicles in each parking space, while in the proposed work, load balancing is based on the count of people for every parking space.

Load balancing and equity distribution were utilized by many researchers in different domains. The authors in^24^ developed upper bounds and lower bounds for the projects distribution across regions. The objective was to ensure reducing the gap between all assigned budget to defined regions. In addition, exact solution was introduced by the authors using the branch-and-bound method. In the same context of the projects distribution problem, the authors in^25^ proposed a mathematical model to solve the proposed problem. To drive solutions with an acceptable execution time, several approximate solutions were developed. For example, the authors in^26^ proposed three heuristics based on the probabilistic and iterative approach. Recently, authors in^27^ proposed five heuristics to solve the problem. While the authors in^28^ employed load balancing to address the problem of used space storage in memory system by developing several approximate solutions to find an appropriate schedule that ensures the fair distribution of the storage system files. The fair distribution ensures the closest gap between storage media in terms of used spaces.

Equity distribution was also used in the domain of networks. For example, the author in^29^ considered the equity distribution problem of the data to be transmitted through the routers. Multi-fit algorithm and a subset-sum approach were built to solve the given problem.

In the aviation domain, for the maximization of the gas turbine engines, several algorithms were proposed to give an appropriate solution for the maximization of the aircraft operating time^30^, where the authors proposed several lower bounds based essentially on the iterative method and the reformulation of the studied problem to a knapsack problem. In the same context many heuristics were presented to address the such problems as in^31^, where the authors developed several approximate solutions based on the randomization method to solve the related problem.

Recently, the author in^32^ proposed the problem of parking lots allocation of different vehicle types to the available parking lots, based on the fair distribution of total number of people in each vaccine center. This work is designed to manage the traffic at COVID-19 vaccine delivery centers, where the author developed nine heuristics to tackle the presented problem. The given results, showed that the C3S algorithm, outperformed other algorithms, with a percentage of 94%. A comparison study was made between C3S algorithm and the algorithms presented in this article to explore the difference in performance and to show the superiority of the algorithms presented in this article over the best algorithms presented in^32^. It is worthy to remark that, the work presented in this article is a generalization of^32^, with algorithms that consider all types of parking based on new architecture and parking process.

The service-oriented computing treated in^33,34^ can be extended and used to be adopted on the studied problem. In addition, the deep learning technics developed in^35,36^ can be used to give an enhanced heuristics for the studied problem. Other techniques can be adopted to the studied problem^37,38^.

Several scheduling algorithms can be adopted on the studied problem to solve the equity distribution of the parking management^39–42^.

Recently, several equity distribution works are treated^43,44^.

## Research gaps

Despite the numerous researches about the problem of allocating available parking spaces, many of these researches suffer from various shortcomings when applied to find appropriate solutions to this problem. The following research gaps are concluded based on the previous works that were analyzed in the literature:

1: Considering the various types of lots that are suitable for all vehicle types.
2: Alleviating the occurrences of traffic jams, which leads to higher energy consumption and longer waiting time to reach the reserved parking space.
3: considering static parking allocation and cannot handle the real-time changes in the available parking spaces i.e. the dynamic parking allocation problem. Thus, it will not prevent vehicle overcrowding in parking spaces.
4: Did not consider the different number of passengers in each vehicle and the overall count of people inside each parking space if it was fully utilized. Moreover, the load balancing presented in the literature was based on the over all count of vehicles in each parking space, while in the proposed work, load balancing is based on the total number of people inside each parking space.

The searches discussed in the literature addressed the allocation of parking lots by developing algorithms and different approaches that ensure providing the vehicles with the required parking lots while considering customer and vendor constraints and at the same time utilizing the parking lots efficiently. But none of these researches consider the number of people inside these vehicles, which may lead to the accumulation of people inside these parking spaces or at the exits of these parking spaces, as this will lead to crowds and unwelcome chaos. The work presented in this context provides the available parking lot for each vehicle while ensuring a fair distribution of parking lots based on the number of people inside each parking space to avoid congestion.

## Problem formulation

This section presents the parking allocation problem for smart cities.

### Notations and problem representation

The major goal of this study, is to build a set of algorithms that will allocate vehicles to the available parking lots in a given parking spaces, based on the count of people in each vehicle to ensure fair distribution of people within each parking space, to minimize the required time for vehicles to reach the required parking lot and in the same time to avoid people overcrowding.

**Figure 1:**
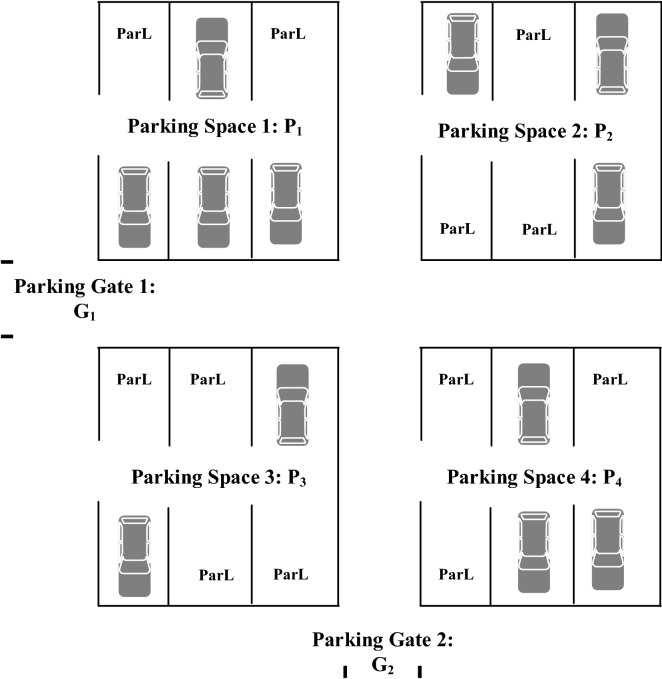
Parking spaces representation example.

In this research it is assumed that parking area consists of a set of parking spaces, and each parking space, has a specified number of parking lots as shown in Fig. 1. Each parking lot is denoted by *ParL*. Each parking area has a set of parking spaces and a set of parking gates that is denoted by $G=\{G_1,\ldots ,G_{n_G}\}$ where $G_k$ is the parking gate number *k*, while the total number of parking gates will be denoted by $n_G$. The set of parking spaces is denoted by *P*. Each parking space is denoted by $P_i$, where *i* is the index of each parking space. All of these details are shown in Fig. 1, where this figure is an example of 4 parking spaces with two parking gates, $G_1$ and $G_2$. Each parking space has 6 parking lots. The total number of parking spaces is denoted by $n_p$.

**Figure 2:**
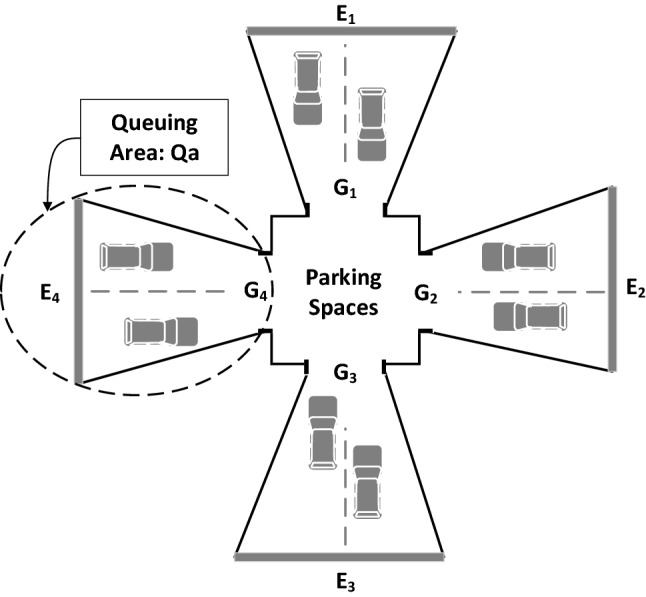
Entrance and queuing area representation.

The main entrance to the parking area, represents the access point to the main gate of the parking spaces and is denoted by $E_k$, while the space between $E_k$ and $G_k$ is denoted by $Qa_k$ which represents the queuing area, as shown in Fig. 2, where this figure is an example of parking spaces that have 4 main entrances. The queuing area is the space where the incoming vehicles are queued, in order to be allocated by the scheduler.

A set of sensing devices will be used to collect data, about people inside each vehicle in the queuing area $Qa_k$ that enters from the main entrance $E_k$. This data is sent to the data control unit to derive people count inside each vehicle, the derived data will be sent to the scheduler to update the scheduling process and reallocate the vehicles based on the updated data.

To ensure the equity distribution constraint, the scheduler will consider the updated total number of people inside each parking space, before allocating any vehicle. Let $Mv_i$ be the maximum number of vehicles that can be allocated to a certain parking space. If the $Mv_i$ is reached, the scheduler will reallocate the vehicle to the first available parking lot in any of the available parking spaces.

This means that the scheduler will consider the $n_p-1$ remaining parking spaces, while ensuring that the equity distribution is not violated. Besides, when the total number of vehicles is greater than $Mv_i$ $\forall i \{1\le i\le nv_k\}$, the control unit will show a message on the display units at the entrance of each queuing area that a certain parking space is full.

The set of vehicles in queuing area $Qa_k$ will be denoted by $V^k$. Each vehicle in the queuing area will be denoted by $V_j^k$ where *j* is the index of each vehicle in the queuing area. The total number of vehicles in each queuing area $Qa_k$ is denoted by $nv_k$. For any period of time, the total number of vehicles in all queuing areas is denoted by *nv*. So, $nv=\sum _{k=1}^{k=n_G}nv_k$. It is worthy to notice that, the number of main entrances $E_k$ equals to the number of parking space gates $n_G$ and is equal to the number of queuing areas.

The number of people in each vehicle $V_j^k$ in queuing area $Qa_k$ is denoted by $Pe_j^k$ and the corresponding set of all $Pe_j^k$ for queuing area $Qa_k$ is denoted by $Pe^k$. At any period of time, the set that contains all $Pe_j^k$ for all queuing areas is denoted by *Pe*. So, $Pe=\{Pe_j^k, \forall 1\le j\le nv_k \text { and }\forall 1\le k\le n_G\}$. To simplify variables presentation, the two indexes *j* and *k* are substituted by *s*, with $1\le s\le nv\}$. Indeed, each element in *Pe* is represented by $Pe_s$. Each element $Pe_s$ will be the number of people of vehicle $V_s$. The set that constituted by all elements $Pe_s$ is denoted by *PS*. So, $PS=Pe$.

The total number of people that is assigned for each parking space $P_i$ is known as the parking space load and is denoted by $Lo_i$. When a vehicle $V_j^k$ is allocated to a parking space $P_i$, the accumulated people count in that parking space is denoted by $Lo_i^{k,j}$.

#### *Remark 1*

$$\sum _{i=1}^{i=n_p}Lo_i=\sum _{k=1}^{k=n_G}\sum _{j=1}^{j=nv_k}Pe_j^k$$

In Remark 1, the overall count of people in all parking spaces is equal to the total number of people in all vehicles for all queuing areas.

The problem here is to properly schedule the given set of vehicles and parking spaces. The objective, is to seek for a schedule that solves the given problem, while ensuring an equitable distribution of people total count, per each parking space. To attain this, reduce variations in the resulted parking spaces load, by minimizing the summation of the differences between each parking space load $Lo_i$ and its minimum load $Lo_{min}$.

The gap value of the people total count, for each parking space, is found as in Eq. (1).

1 $$\begin{aligned} g=\sum _{i=1}^{i=n_p}[Lo_i-Lo_{min}] \end{aligned}$$

In this paper, the main objective is the minimization of *g*. In addition, for each parking space, the equitable people distribution must be guaranteed.

### Mathematical model

The requirements to formulate the smart parking model consider the definition of the problem constraints. The model was formulated to present a solution to the problem of smart parking scheduling for smart cities. The focus was on deciding which parking space will be allocated to a particular vehicle. Moreover, Sets, parameters, objective variables function, and restrictions were defined.

#### Sets

The model has two sets *P* and *PS*.

#### Parameters

The parameter to be considered in the presented model is the number of people $Pe_s$ in vehicle $V_s$ $\forall s$ $\{1\le s\le nv\}$.

#### Variables

2 $$\begin{aligned} x_{is}= {\left\{ \begin{array}{ll} 1 &{} \text {if vehicle } s \text { is assigned to parking } i,\\ 0 &{} \text {Otherwise}.\\ \end{array}\right. } \end{aligned}$$

#### Objective function

3 $$\begin{aligned} \min \sum _{i=1}^{i=n_p}[Lo_i-Lo_{min}] \end{aligned}$$

The objective function stated that the calculated gap *g* should be minimized.

#### Constraints

4 $$\begin{aligned}&\sum _{i=1}^{n_p} x_{is}=1, \forall s\in \{1,\ldots ,nv\} \end{aligned}$$

5 $$\begin{aligned}&\sum _{s=1}^{nv} Pe_sx_{is} \ge Lo_{min}, \forall i\in \{1,\ldots ,n_p\} \end{aligned}$$

6 $$\begin{aligned}&x_{is}\in \{0,1\},\forall i\in \{1,\ldots ,n_p\} \text { and } \forall s\in \{1,\ldots ,nv\} \end{aligned}$$

7 $$\begin{aligned}&Lo_{min} > 0 \end{aligned}$$

Restriction in Eq. (4) ensures that each vehicle *s* can only be assigned to a single parking space *i* of *nv* available parking spaces. Restriction in Eq. (5) establishes the minimum parking space load ($Lo_{min}$) as the smallest amount of number of people on each parking space. Restriction in Eq. (6) defines the nature of the decision variable, being of binary type. Restriction in Eq. (7) establishes the non-negativity of the variables.

##### *Example 1*

This example shows vehicles scheduling for different parking spaces by applying any given algorithm, assuming that we have two parking spaces and four queuing areas. The people count per each vehicle in this example, is shown in Table 1.

**Table 1:** $Pe_j^k$ values for each $Qa_k$ and each $V_j^k$.

*k*/*j*	1	2	3	4	5	6
1	3	2	4	5	2	–
2	5	4	1	–	–	–
3	3	2	1	–	–	–
4	4	3	2	4	1	1

The symbol (–) in Table 1 means that the number of vehicles in that queuing area is less than *j*. For this example, in queuing area $Qa_1$ there are five vehicles, while in queuing area $Qa_2$ there are three vehicles, in queuing area $Qa_3$ there are three vehicles and in queuing area $Qa_4$ there are six vehicles. Therefore, the total number of vehicles for this example is 17 vehicles. The objective here is to fairly distribute the given number of people to the given parking spaces.

**Figure 3:**
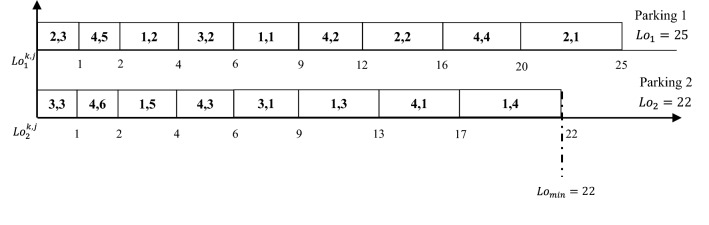
Scheduling of vehicles to parking spaces in Example 1.

This problem can be formulated in two sets; the first set has the 17 vehicles with different count of people in each vehicle. The second set has the two parking spaces. So, the problem here is to schedule the elements of the first set to the elements of the second set. Applying any algorithm that can sort the vehicles in a decreasing order based on the count of people in each vehicle, will result the schedule shown in Fig. 3.

As shown in Fig. 3, after applying the algorithm on the above instances the load in parking space 1 is $Lo_1=25$ and in parking space 2 is $Lo_2=22$. So, applying Eq. (1), the load gap between parking space 1 and parking space 2 is $g=Lo_1-Lo_2=25-22=3$. The objective is to develop an algorithm that can minimize the obtained load gap *g*. For this example, the objective is to a schedule with *g* value less than 3.

##### **Proposition 1**

*The studied problem is an NP-hard problem*.

##### *Proof*

The studied problem can be formulated as an identical and parallel processors scheduling problem. The correspondence between the studied problem and the parallel processors scheduling problem can be formulated as follows: The processors are represented by the parking spaces, while tasks are represented by vehicles. Finally, processing time is represented by the number of people inside each vehicle. The parallel processors scheduling problem with the goal of maximizing the minimum completion time ($C_{min}$) is proofed in literature as an NP-hard problem. In other hand the objective in this research is to minimize *g* which is equal to $\sum _{i=1}^{i=n_p}[Lo_i-Lo_{min}]$ and is equal to $\sum _{i=1}^{i=n_p}Lo_i-\sum _{i=1}^{i=n_p}Lo_{min}$.

Based on Remark 1, $g=\sum _{k=1}^{k=n_G}\sum _{j=1}^{j=nv_k}Pe_j^k- \sum _{i=1}^{i=n_p}Lo_{min}$. The number $\sum _{k=1}^{k=n_G}\sum _{j=1}^{j=nv_k}Pe_j^k$ is fixed and does not change based on the used algorithms. Then the problem will be equivalent to minimizing $- \sum _{i=1}^{i=n_p}Lo_{min}$ which is equivalent to maximizing $\sum _{i=1}^{i=n_p}Lo_{min}$ that is corresponding to maximizing the minimum completion time ($C_{min}$) for the parallel processors scheduling problem. Since the latter problem is NP-hard, the presented problem is NP-hard.

$$\square$$

## The developed algorithms

Seven algorithms are detailed in this section. These algorithms are based on four techniques. Iterative method that selects an iteration number to repeat a certain procedure in order to select the best solution, is the first technique. The second one is the randomization approach, where a probability value is applied to choose between one vehicle or one parking. Probability value varies according to the chosen algorithm. The third technique is a combination of the two previous techniques. Each combination will give a new algorithm with new results.

The fourth technique is the solution of the two parking problem using the subset-sum problem. The solution of the sub-set problem is inspired by the two parallel machines problem solved using the sub-set problem. Indeed, from the latter work, we call the procedure of sub-set problem to solve the two parking spaces problem. These parking spaces are the most loaded and the least loaded.

In the next subsection, we will use the non-increasing order based procedure denoted by NI. This procedure is based on the following strategy: initially, sort all the vehicles in non-increasing order according to the number of people inside it. The second step is to assign the vehicle that has the greatest number of people to the parking space that has the minimum total number of people. After that, continue in this manner of scheduling until all vehicles are scheduled.

**Figure 4:**
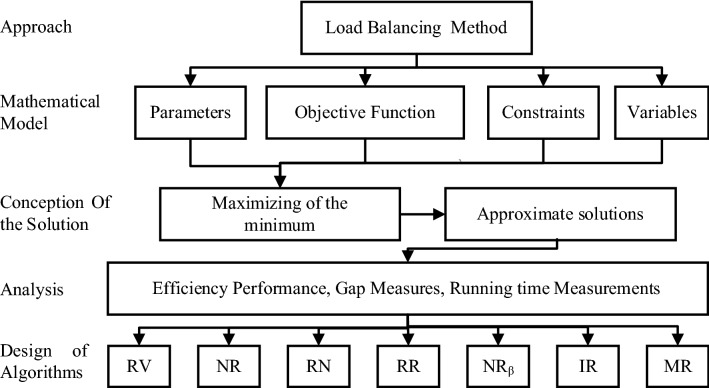
Logical structure diagram.

Figure 4 shows the logical structure diagram. To solve the discussed problem, this research started by collecting the required data, which was analyzed to derive the required parameters. These parameters were used to specify the needed constraints, then to derive the variables and specify the objective functions to obtain maximizing of the minimum to reach the approximate solutions. The obtained solutions will be used to measure the performance metrics, efficiency, and running time calculations. These calculations are the basis to design the developed algorithms that will be the framework of the proposed approach.

### Iterative random vehicle algorithm (RV)

Randomization method is utilized to develop this algorithms as follows. First, classify the vehicles into three types. The first type, vehicle is chosen to be scheduled based on vehicle index. The next type is the scheduling of vehicles based on the non-decreasing order of the count of people in the vehicle. The third type is the vehicle scheduling based on the decreasing order of the people count for each vehicle. For a certain type, choose a vehicle randomly from the set of given vehicles. After that, allocate the selected vehicle to the parking space that has the minimum total number of people, then repeat until finishing all vehicles. This process is being repeated for many times. Therefore, for each type, execute the selection of vehicles for *lim* times.

In this context, the function rand(*a*, *b*) is responsible of deriving integers in the range *a* and *b*, while Asg(*j*) is the function that assigns the vehicle *j* to the parking that has the minimum number of people. Let In() be the function that sorts the given vehicles in an increasing order based on the number of people inside it. While, De() be the function that sort the given vehicles in a decreasing order based on the number of people inside it. For RV algorithm the iterations number *lim* is fixed to 1000. This algorithm is denoted by RV and the related execution steps are described in the algorithm in Table 2.

**Table 2:**
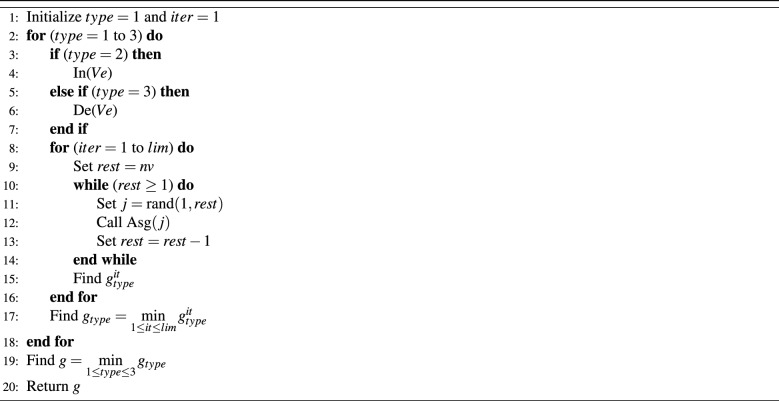
Iterative random vehicle algorithm (RV).

### M-vehicles with NI and random choice algorithm (NR)

This algorithm works as follows, schedule part of the vehicles using the NI algorithm, then the remaining vehicles are scheduled by applying the random choice of any of the remaining vehicles. The first chosen part is prepared based on a multiplication by the number of parking spaces, which is called the multiplier and is denoted by *M*. To illustrate, apply the NI algorithm for the first $2\times n_p$ vehicles to be scheduled, the rest of the vehicles will be chosen randomly and will be allocated to the parking space that has the minimum number of people. For this case, the multiplier *M* is equal to 2. Iterate this algorithm for *lim* times. After that increment the multiplier *M* to 3 and so on until $M\times n_p<50$ and $M\times n_p<nv$. This algorithm is given the name NR and Table 3 describes the related execution steps.

**Table 3:**
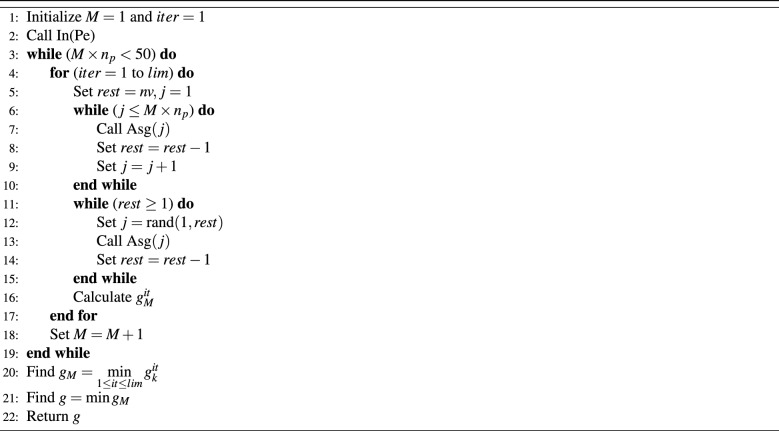
M-vehicles with NI and random choice algorithm (NR).

### M-vehicles with randomized-NI and NI algorithm (RN)

This algorithm works as follows, schedule part of the vehicles using the randomized-NI algorithm, then the remaining vehicles are scheduled by applying the NI algorithm. The first chosen part is performed based on a multiplication by the number of parking spaces, which is called the multiplier and is denoted by *M*. The same iteration which is based on the multiplier *M* adopted for NR will be utilized in this algorithm. This algorithm will be denoted by RN.

In the randomized-NI procedure, the randomization is achieved by selecting a probability $\alpha$ to choose vehicle with the largest count of people and with $1-\alpha$ for the next vehicle with the largest number of people . The algorithm given in Table 4 describes the instructions of the randomized-NI procedure RNI(.). In this algorithm $M\times n_p$ (the input of the procedure) is the set of vehicles that will be set by the multiplier *M* described in NR.

**Table 4:**
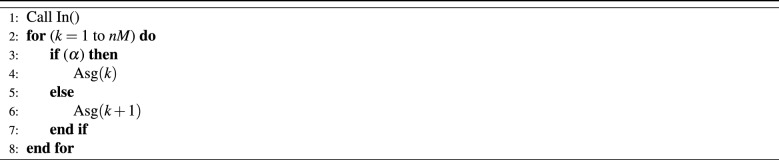
Randomized-NI function (RNI(*nM*)).

Next, the instructions that elaborate RN as detailed in the algorithm illustrated in Table 5 is given.

**Table 5:**
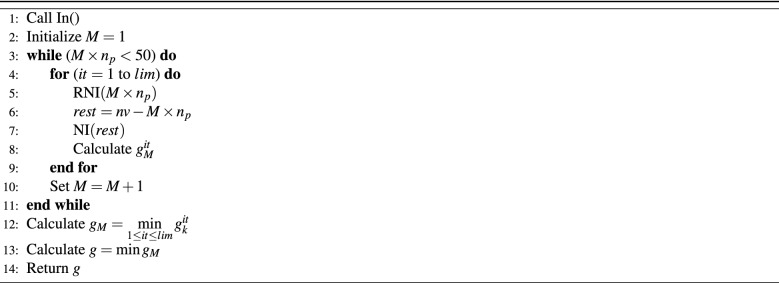
M-vehicles with randomized-NI and NI algorithm (RN).

### M-vehicles with randomized-NI and random vehicle algorithm (RR)

This algorithm works as follows, schedule part of the vehicles using the randomized-NI algorithm as described in the “M-vehicles with randomized-NI and NI algorithm (RN)” section, then schedule the remaining vehicles by applying the random choice of any of the remaining vehicles. The same iteration which is based on the multiplier *M* adopted for NR will be used in this algorithm. This algorithm is denoted by RR.

### Part of vehicles with NI and random vehicle algorithm ($\hbox {NR}_\beta$)

This algorithm works as follows, schedule part of the vehicles using the NI algorithm, then schedule the remaining vehicles by applying the random choice of any of the remaining vehicles. This algorithm will introduce the percentage that will be used to divide the set of given vehicles. First, define $\beta$ to be the probability that will be used to apply the division. Then, after applying this division, two subsets $S_1$ and $S_2$, will be generated. The $nv\times \beta$ first vehicles, will constitute the subset $S_1$ and the remaining vehicles will constitute the subset $S_2$. The next step is to apply the NI algorithm for $S_1$ then apply the random vehicles choice for $S_2$. Experimentally, $\beta$ value is in the range of $[0.1-0.9]$, with step of 0.1. For all $\beta$ values, iterate the algorithm for *lim* times.

### Iterative randomized-NI algorithm (IR)

This algorithm works as follows, choose randomly between the two vehicles that has the largest number of people. Indeed, probability $\sigma$ will be applied to choose the most loaded vehicle and the probability $1-\sigma$ is applied to choose the second most loaded vehicle. The probability value of is changed several times. In this algorithm, probability values are in the range of $\{0.1,0.2,0.3,0.4,0.5,0.6,0.7,0.8,0.9\}$. The algorithm is repeated for 1000 times, then the best solution is selected.

### Multi-repeating randomized-NI and subset-sum solution algorithm (MR)

This algorithm works as follows. First, let $\hbox {IR}_{0.4}$ be the algorithm IR with a fixed value of probability of 0.4. Next, the most loaded parking space and the least loaded parking space will be selected. The list that has the vehicles constituted by these latter parking spaces is denoted by Ls. The cardinality of Ls is denoted by $n_L$. After that, call the subset-sum procedure to solve the two parking problem, with Ls containing the number of people for each vehicle and $n_L$ the number of vehicles. A new schedule of two parking will be obtained by the solution of subset-sum. This new schedule will be applied to the two parking spaces, the new *g* value will be calculated and the best solution will be selected. Repeat the IR algorithm call for 40 times and for each call, a subset-sum procedure is applied. The subset-sum procedure applied on list X and number of elements y, is denoted by SS(X,y). After completing all iterations, the best solution is selected. All of these instructions are shown in the algorithm illustrated in Table 6.

**Table 6:**
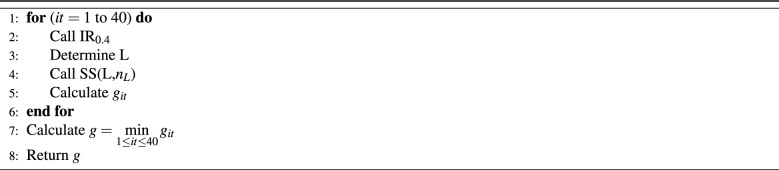
Multi-Repeating randomized-NI and subset-sum solution algorithm (MR).

## Experimental results and discussion

The performance measurement of the developed algorithms will be displayed in this section. The proposed algorithms were compared against the best algorithms in literature, the comparison is detailed by inclusion of C3S algorithm in all tables. This section is organized as follows. The used materiel is presented. After that, the used data set is detailed and shown. The evaluation metrics are presented to be used for the assessment of the proposed algorithms comparing with the best one from literature C3S. Finally, the results analysis and discussion are detailed using four tables illustrated after implementation of all proposed algorithms and the best algorithm from literature.

### Used materiel and environment

The programming language used to implement the developed algorithms is Microsoft Visual C++. The computer that was used to execute the programs of the proposed algorithms has an Intel(R) Core (TM) i7-3770CPU @3.40GHz and 8GB RAM.

### Dataset description

The number of people $Pe_s$ is generated using the uniform distribution denoted by *U*(.). In this paper 9 classes of instances were adopted as follows:

- Class 1: 80% of $Pe_s$ are generated according to *U*(6, 15) and 20% of $Pe_s$ are generated according to *U*(20, 40).
- Class 2: 80% of $Pe_s$ are generated according to *U*(1, 9) and 20% of $Pe_s$ are generated according to *U*(20, 50).
- Class 3: 80% of $Pe_s$ are generated according to *U*(1, 9) and 20% of $Pe_s$ are generated according to *U*(30, 70).
- Class 4: 10% of $Pe_s$ are generated according to *U*(6, 15) and 90% of $Pe_s$ are generated according to *U*(20, 40).
- Class 5: 10% of $Pe_s$ are generated according to *U*(1, 9) and 90% of $Pe_s$ are generated according to *U*(20, 50).
- Class 6: 10% of $Pe_s$ are generated according to *U*(1, 9) and 90% of $Pe_s$ are generated according to *U*(30, 70).
- Class 7: 100% of $Pe_s$ are generated according to *U*(20, 40).
- Class 8: 100% of $Pe_s$ are generated according to *U*(20, 50).
- Class 9: 100% of $Pe_s$ are generated according to *U*(30, 70).

In this paper, many values of the pair $(nv,n_p)$ were used as Table 7 displays.

**Table 7:** Generation of $(nv,n_p)$.

*nv*	$n_p$
10	2, 3, 4
20	2, 3, 4, 5
50, 100, 200, 500	2, 3, 4, 5, 6

For each tuple $(nv,n_p,class)$, the instances of people count that will be generated for the calculation will be 10 different instances. As a result, the overall generated instances are, $(3+4+4\times 5)\times 10\times 9=2430$.

### Evaluation metrics

To evaluate the performance of the given algorithms, the following metrics are given, as follows:

- $B^*$: Returns the best value among all algorithm values, obtained after the execution of all algorithms.
- *B*: Returns the values of the current algorithm (the evaluated algorithm).
- *Per*: The instances percentage where $B=B^*$.
- $$G=\frac{B-B^*}{B}$$, if $$B=0$$ then $$G=0$$.
- *AVg*: The average of the gap *G* for a given number of instances.
- *Time*: Execution time in seconds, or the result of “-” if the execution time is less than 0.001 s.

### Results analysis and discussion

In this subsection, we present several analysis and discussion of the presented algorithms and compare the proposed algorithms with the best one obtained in literature which is C3S^32^. First, we present an aver of all results illustrated in Table 8. After that, a comparison of average gap and average execution time according to *nv*, $n_p$, and classes are presented in Tables 9, 10, and 11.

Table 8 presents the overall results of all the developed algorithms. Based on the shown results, it can be concluded that MR is the best algorithm, with a percentage of 96.1 %, and $AVg=0.02$, and an average execution time of 0.007 s. Comparing with C3S which has a percentage of 92.7 % and a gap of 0.06, and an execution time of 0.020 s. It is obvious that, the best proposed algorithm produces a better solution comparing C3S algorithm. The $\hbox {NR}_\beta$ percentage of 88.1% makes it the second best algorithm. The algorithm that has the lowest percentage is RV with a value of 64.5 %.

**Table 8:** Overall results of all algorithms.

	RV	NR	RN	RR	$\hbox {NR}_\beta$	IR	C3S	MR
*Perc*	64.5%	72.1%	73.1%	73.5%	88.1%	78.2%	92.7%	96.1%
*AVg*	0.29	0.22	0.23	0.21	0.10	0.19	0.06	0.02
*Time*	0.053	0.157	0.104	0.165	0.172	0.154	0.020	0.007

Table 9 presents *AVg* and *Time* variations based on *nv* for the given algorithms. In these results, average gap values less than 0.001 is returned by algorithms RV and $\hbox {NR}_\beta$ when $nv=10$ and by algorithm MR when $nv=500$. The maximum *AVg* of 0.37 is returned by RV when $nv=200$. The average gap obtained by MR is always better then obtained by C3S for all values of *nv* excepting $nv=10$. The remarkable amelioration of gap comparing with literature is obtained when $nv=50$. Indeed, the average gap of C3S is 0.10 and for MR is 0.01. The maximum average execution time of 0.526 s is reached by the algorithm $\hbox {NR}_\beta$ when $nv=500$. However, the algorithm MR showed an excellent average execution time of values with maximum 0.025 s which is obtained when $nv=500$. Comparing with C3S, the maximum average execution time is 0.069 s which is obtained when $nv=500$. For all algorithms in Table 9, it can be noticed from the given results that, when the number of vehicles increases the average execution time increases.

**Table 9:** *AVg* and *Time* variations according to *nv* for all algorithms.

*nv*	RV		NR		RN		RR		$\hbox {NR}_\beta$		IR		C3S		MR	
	*AVg*	*Time*	*AVg*	*Time*	*AVg*	*Time*	*AVg*	*Time*	*AVg*	*Time*	*AVg*	*Time*	*AVg*	*Time*	*AVg*	*Time*
10	0.00	0.003	0.19	0.001	0.22	0.001	0.14	0.002	0.00	0.007	0.22	0.005	0.02	0.001	0.07	0.001
20	0.24	0.006	0.22	0.004	0.18	0.006	0.18	0.014	0.13	0.030	0.18	0.015	0.07	0.002	0.04	0.001
50	0.33	0.019	0.19	0.057	0.28	0.065	0.17	0.076	0.16	0.081	0.26	0.061	0.10	0.005	0.01	0.001
100	0.36	0.053	0.23	0.106	0.25	0.083	0.24	0.117	0.10	0.109	0.16	0.121	0.05	0.010	0.02	0.002
200	0.37	0.075	0.24	0.187	0.23	0.115	0.24	0.199	0.09	0.187	0.15	0.240	0.05	0.022	0.01	0.006
500	0.33	0.131	0.25	0.493	0.20	0.295	0.27	0.488	0.07	0.526	0.15	0.393	0.05	0.069	0.00	0.025

Table 10 presents *AVg* and *Time* variations according to $n_p$ for all algorithms. When number of parking spaces $n_p$ increases, the average gap increases for all algorithms except for RN at $n_p=\{4,5\}$, IR at $n_p=\{4,5\}$, and MR at $n_p=6$. The average gap values of less than 0.001 is obtained by four algorithms among the proposed ones. Indeed, this result is obtained by algorithm RV, RR, and MR when $n_p=2$ and by algorithm $\hbox {NR}_\beta$ when $n_p=\{2,3\}$. The average gap values of $\hbox {NR}_\beta$ was in the range of less than 0.001 to 0.40. Indeed, the best average gap of less than 0.001 was obtained at $n_p=\{2,3\}$ while the maximum *AVg* of 0.40 was obtained when $n_p=6$. Surprisingly, the worst average execution time of 0.240 s is obtained by the algorithm $\hbox {NR}_\beta$ algorithm where $n_p=6$.

**Table 10:** *AVg* and *Time* variations according to $n_p$ for all algorithms.

$n_p$	RV		NR		RN		RR		$\hbox {NR}_\beta$		IR		C3S		MR	
	*AVg*	*Time*	*AVg*	*Time*	*AVg*	*Time*	*AVg*	*Time*	*AVg*	*Time*	*AVg*	*Time*	*AVg*	*Time*	*AVg*	*Time*
2	0.00	0.046	0.01	0.217	0.01	0.138	0.00	0.230	0.00	0.148	0.01	0.141	0.00	0.014	0.00	0.003
3	0.01	0.046	0.05	0.159	0.36	0.105	0.02	0.168	0.00	0.150	0.24	0.133	0.01	0.017	0.02	0.006
4	0.28	0.049	0.23	0.129	0.22	0.086	0.22	0.135	0.07	0.158	0.20	0.137	0.04	0.019	0.04	0.005
5	0.63	0.058	0.39	0.124	0.12	0.085	0.40	0.132	0.13	0.192	0.09	0.166	0.05	0.024	0.04	0.007
6	0.75	0.074	0.58	0.148	0.54	0.105	0.57	0.148	0.40	0.240	0.48	0.213	0.27	0.030	0.01	0.014

Table 11 presents *AVg* and *Time* variations according to classes for all algorithms. The first notice on the given results is that, changing a class has no effect on the average execution time of the developed algorithms. However, changing a class has an effect on the average gap for all algorithms excluding MR. Indeed, for classes 1,2, and 3 the average gap has the same range but for all others classes the range will be more remarkable. For algorithm MR, the maximum *AVg* of 0.04 is gained for class 5, while for C3S the maximum *AVg* of 0.18 is gained for class 9.

**Table 11:** *AVg* and *Time* variations according to classes for all algorithms.

Class	RV		NR		RN		RR		$\hbox {NR}_\beta$		IR		C3S		MR	
	*AVg*	*Time*	*AVg*	*Time*	*AVg*	*Time*	*AVg*	*Time*	*AVg*	*Time*	*AVg*	*Time*	*AVg*	*Time*	*AVg*	*Time*
1	0.17	0.056	0.09	0.154	0.11	0.104	0.07	0.171	0.02	0.174	0.06	0.157	0.01	0.020	0.02	0.005
2	0.10	0.056	0.01	0.167	0.00	0.103	0.02	0.176	0.00	0.178	0.00	0.163	0.00	0.020	0.02	0.006
3	0.20	0.054	0.01	0.170	0.00	0.106	0.02	0.174	0.00	0.179	0.00	0.153	0.00	0.020	0.00	0.007
4	0.31	0.055	0.27	0.155	0.37	0.106	0.26	0.159	0.18	0.170	0.22	0.150	0.04	0.021	0.03	0.005
5	0.34	0.054	0.30	0.155	0.20	0.101	0.28	0.162	0.07	0.174	0.16	0.149	0.03	0.020	0.04	0.006
6	0.41	0.053	0.35	0.158	0.25	0.105	0.34	0.163	0.13	0.171	0.16	0.155	0.06	0.021	0.03	0.006
7	0.32	0.048	0.27	0.151	0.38	0.103	0.24	0.160	0.13	0.167	0.36	0.152	0.12	0.020	0.02	0.008
8	0.38	0.050	0.32	0.150	0.36	0.106	0.30	0.161	0.14	0.165	0.33	0.150	0.10	0.020	0.03	0.007
9	0.42	0.049	0.38	0.153	0.41	0.104	0.37	0.159	0.19	0.173	0.37	0.154	0.18	0.020	0.02	0.009

In light of the foregoing discussion and based on the obtained results, the application of the proposed approach may contribute significantly to the parking allocation problem solving, especially in large and crowded cities or in areas that witness huge human gatherings, such as central commercial sites, sports stadiums, and activity yards for various social activities, as well as airports. Mayors, governors, and city managers, whether in ordinary or smart cities, can take advantage of the proposed approach to achieve the optimal use of parking lots and to solve many parking allocations problems, such as avoiding crowding of people, minimizing waiting time, and saving fuel. Furthermore, an available parking lot facilitates people’s access to their destinations, whether for work, commerce, entertainment, etc. Adopting the proposed model when designing parking spaces will contribute significantly to increasing the effectiveness of the proposed algorithms and thus maximizing the desired results when utilizing these algorithms, as it will reduce the number of people roaming the streets of different cities in search of parking lots for their vehicles. The form and modus operandi of modern parking lots will also be changed based on the results obtained from the ongoing research in this regard.

## Conclusion and prospects

### Conclusion

This research provides a solution to the problem of equitable distribution of parking lots in parking areas within smart cities for different vehicle types. Each parking area will be divided into parking spaces and each parking space has a set of known parking lots. People equity distribution for all parking spaces is the major goal of this work which was achieved by a set of developed algorithms that allocate parking lots to vehicles of different types. Vehicles are forwarded to a certain parking space based on the overall number of people inside it. The total number of people inside each parking space specifies the number of available parking lots. In this paper, seven algorithms were developed to solve the studied problem. Experimental results showed that algorithm MR reached a remarkable percentage of 96.%, *AVg* value of 0.02, and an average execution time of 0.007 s, making it better than the C3S algorithm. The $\hbox {NR}_\beta$ algorithm with a percentage of 88.1 % was the second-best algorithm. The obtained results showed that the developed algorithms succeeded in minimizing the gap in the total number of people for all parking spaces, controlling the total number of people in all parking spaces, avoiding congestions, and reducing the time needed for people to reach their destinations.

### Prospects

In the future four aspects will be addressed . Expand the list of the used classes and carry out more detailed discussions of the presented algorithms compared to the main methods presented in the literature with the goal of offering academics and researchers more directions on how to address the parking management problem in smart city. Second, we wish to verify the efficiency and capability of the developed algorithms offered through this review. Third, we intend to conduct a study to examine the possibility of hybridizing different proposed algorithms in order to achieve better performance in terms of execution time and gap. Fourth, the proposed algorithms can be enhanced after testing them as an initial solution by several meta-heuristics and can be used by exact methods to determine the exact solution.
